# Exploring the Factor Structure of Criminogenic Cognitions in Incarcerated Males: Psychometric Evaluation of the Criminogenic Cognitions Scale (CCS)

**DOI:** 10.3390/ejihpe15030027

**Published:** 2025-02-21

**Authors:** Teresa Pereira, Catarina Oliveira, Miguel Basto-Pereira

**Affiliations:** William James Center for Research, Ispa-Instituto Universitário, 1149-041 Lisbon, Portugal; mbpereira@ispa.pt (T.P.); catarina.26.2000@gmail.com (C.O.)

**Keywords:** criminogenic cognitions, exploratory factor analysis, incarceration, inmates, psychometric properties, forensic assessment

## Abstract

Despite the importance of criminogenic thinking in addressing criminal behavior, validated instruments to measure these cognitions in Portuguese forensic settings are scarce. Therefore, the main aim of this study was to explore the psychometric properties of the Criminogenic Cognitions Scale (CCS) in a sample of 364 Portuguese incarcerated males (*M*_age_ = 37.88, *SD* = 10.88). An exploratory factor analysis was conducted, indicating a 15-item, two-factor structure (KMO = 0.82; Bartlett’s test, χ^2^ = 1841.2, *df* = 105, *p* < 0.001). The CCS dimensions, comprising Short-Term Orientation and Responsibility Evasion and Authority Resistance, demonstrated satisfactory psychometric properties, including convergent validity with antisocial traits, moral disengagement, and self-control dimensions, as well as internal consistency (omega coefficient = 0.60–0.77; composite reliability = 74–91; coefficient H = 89–95), and sensitivity of items. The CCS is a valuable tool within prison settings for assessing criminogenic thinking patterns, supporting risk assessment, the development of targeted rehabilitation programs, and monitoring cognitive changes over time to reduce recidivism, thereby promoting inmates’ safer reintegration into society. Overall, our findings suggest the CCS is a promising tool for assessing criminogenic cognitions in the forensic Portuguese population.

## 1. Introduction

Walters (2019) defined criminal thinking as “a set of attitudes or beliefs connected to criminal behavior that support and maintain a criminal lifestyle” (p. 637). Criminal thinking has been the focus of many studies over the years (e.g., Walters, 2020). Moreover, it encompasses not only what individuals engaged in antisocial and criminal behavior think but also how they think (Vrućinić, 2019; Walters, 2019). Indeed, criminal thinking has displayed correlations with almost all relevant criminal outcomes, such as arrests and juvenile and adult justice involvement (Walters, 2020). Also, criminal thinking has been associated with recidivism (Mitchell & Tafrate, 2012), and decreases in criminal thought patterns have been linked to a reduction in disciplinary infractions and reduced instances of misconduct within incarcerated populations (Folk et al., 2016; Walters, 2017a). More importantly, criminal thinking is inversely related with attitudes towards rehabilitation and social integration (Bartholomew et al., 2018).

Bourke et al. (2013) emphasized the importance of criminogenic cognitions for criminal thinking. In particular, criminogenic cognitions are recognized as crucial for understanding the development and persistence of criminal behavior. Therefore, criminogenic cognitions can be defined as a system that provides justification and rationalization for antisocial behavior (Walters, 2002), characterized by poor decision-making abilities in potentially dangerous situations, which can heighten the risk of committing crimes and increase the likelihood of recidivism (Rivera & Veysey, 2018; Walters, 2020).

In this line, psychological intervention and assessment in correctional facilities targeting criminogenic cognitions are essential for the rehabilitation and social reintegration of inmates, as well as for the safety of society. These interventions primarily aim to promote behavioral change, reduce criminal recidivism, and reintegrate inmates into the community. In this regard, the use of psychological assessment tools, such as the Criminogenic Cognitions Scale (CCS), allows for the identification of individual needs and the implementation of targeted interventions, while also informing the development of policies and strategies that promote mental health in prison contexts. However, the absence of standardized and validated tools for assessing inmates’ risk factors and needs can lead to inconsistent results and a limited understanding of individual needs, hindering the implementation of assessment and intervention programs and compromising their effectiveness.

Social–cognitive theoretical models have played a crucial role in the study of moral development, emphasizing that individuals encounter a variety of unique opportunities and life experiences (Bandura, 2001; Fontaine & Dodge, 2009; Gannon, 2009; Gibbs, 2003). These experiences shape individuals’ behavior based on their interpretations of events, which are aligned with the organization of their beliefs in memory. Research based on these theoretical models has shown that social cognition impacts the likelihood of whether someone displays aggression or violence and has demonstrated strong associations between criminal thinking and a variety of antisocial behaviors in adolescence and adulthood (Caprara et al., 2014).

The core of delinquency is based on a set of values that represent an inversion of the principles upheld by the legal system and society at large (Bilsky & Hermann, 2016). Thus, the justifications for antisocial behavior by individuals who have committed offenses, which are comprehensible to themselves but not to the justice system and society, help them avoid self-blame and mechanisms of social control (Maruna & Copes, 2005). Within this scope, Bandura et al. (1996) introduced a similar concept known as “moral disengagement”, which is one of several variables associated with criminogenic cognitions. Moral disengagement is a social–cognitive mechanism that allows people to detach themselves from moral standards to commit acts that violate moral principles and social norms without feeling guilt and shame, sustaining and being reinforced by criminal thinking (Bandura, 2016; Walters, 2020). Furthermore, studies have shown the associations between moral disengagement and several antisocial behaviors (Bandura, 2016; Gini et al., 2014; Paciello et al., 2008).

Taking into account the work of Yochelson and Samenow (1976), Walters (1995, 2003, 2006a) developed one of the most important theoretical models in the field of criminal thinking. According to Walters’s theory, crime is a lifestyle associated with a system of criminal beliefs and attitudes that include implicit justifications and rationalizations for criminal conduct (Boduszek & Hyland, 2012). This author suggested that criminal thinking can be divided into two categories: criminal thought process and criminal thought content. First, criminal thought process concerns how the individual involved in the crime thinks, taking into account both proactive and reactive dimensions (Walters, 2020). The proactive dimension encompasses cognitive processes of planning and instrumental aggression, while the reactive dimension incorporates patterns of impulsive, irresponsible, and emotional thinking (Walters, 2017b). The second category, criminal thought content, reflects the thoughts of a person involved in the crime, comprising three dimensions: negative attitudes toward authority, positive attitudes toward deviance, and criminal identity (Walters & Morgan, 2018).

According to Andrews and Bonta (2010), antisocial cognition is one of the “central four” domains of risk factors associated with the maintenance of criminal behavior. Antisocial cognition includes “attitudes, values, beliefs, and rationalizations supportive of crime and cognitive emotional states of anger, resentment, and defiance” (Andrews & Bonta, 2010, p. 288). Therefore, questionnaires have been created and tested to measure criminogenic cognitions. Some of the most notable are the Psychological Inventory of Criminal Thinking (PICTS; Walters, 2002), the Texas Christian University Criminal Thinking Scale (TCU-CTS; Knight et al., 2006), and the Criminogenic Thinking Profile (CTP; Mitchell & Tafrate, 2012).

The PICTS is an 80-item self-report measure designed to assess the eight criminal thinking scales: Mollification, Cutoff, Entitlement, Power Orientation, Sentimentality, Superoptimism, Cognitive Indolence, and Discontinuity (Walters, 2002, 2012). It also comprises two validity scales (Confusion and Defensiveness), as well as Reactive and Proactive composite scales (Walters, 2006b). Research employing the PICTS has shown that it predicts recidivism and disciplinary problems in incarcerated people (Walters, 1996, 2009).

The TCU-CTS developed by Knight et al. (2006) is a 37-item measure with six subscales: Entitlement, Justification (mollification), Power Orientation, Personal Irresponsibility (blaming others), Cold Heartedness, and Criminal Rationalization (negative attitude toward law and authority). Although the TCU-CTS initially showed strong reliability, the authors did not present data on validity. Thus, studies subsequently provided mixed support for the validity of the TCU-CTS (Dembo et al., 2007; Taxman et al., 2011). TCU-CTS scores displayed a small correlation with established predictors of recidivism (Tangney et al., 2012).

The CTP is a 62-item self-report instrument with eight thinking-pattern subscales: Disregard for Others, Demand for Excitement, Poor Judgment, Emotionally Disengaged, Parasitic/Exploitive, Grandiosity, Inability to Cope, and Justifying (Jones et al., 2021; Mitchell & Tafrate, 2012). This instrument has shown positive associations with measures of psychopathy and other aggressive personality traits. However, the CTP does not have data regarding its association with institutional misconduct or subsequent offenses (Tangney et al., 2012).

### Validity and Reliability

In this line, Tangney et al. (2002) designed a short instrument, with the objective of assessing criminogenic cognitions across key dimensions. Drawn from the perspectives of frontline rehabilitation professionals, the CCS (Tangney et al., 2002, 2012) is a 25-item self-report measure designed to evaluate the level of criminogenic cognitions across five related dimensions: Notions of Entitlement (“When I want something, I expect people to deliver”), Failure to Accept Responsibility (“Bad childhood experiences are partly to blame for my current situation”), Short-Term Orientation (“The future is unpredictable and there is no point planning for it”), Insensitivity to the Impact of Crime (“A theft is alright as long as the victim is not physically injured”), and Negative Attitudes Toward Authority (“People in positions of authority generally take advantage of others”).

To assess the reliability and validity of the CCS and its dimensions, data were collected from a longitudinal study in the United States, involving 552 felony inmates (Tangney et al., 2012). The CCS demonstrated satisfactory psychometric properties. Regarding internal consistency, the CCS showed values of α = 0.81 for the total score, and αs between 0.61 and 0.75 for its dimensions. This study showed negative correlations between the CCS total score and proneness to guilt, empathy, and self-control. In contrast, the CCS total score was positively correlated with all the indicators of jail infractions. Regarding predictive validity, analysis of receiver operating characteristics curves revealed moderate predictive precision for the CCS total score in relation to the indices of institutional misconduct. In addition, the CCS scores were also linked to a history of criminal activity and to concomitant indicators of aggression, antisocial personality, assessments of psychopathy, and risk for violence. However, despite these findings, the factor structure of the CCS was not tested in the aforementioned study.

Over the years, some studies have used the CCS in different countries, including Pakistan and Romania. Jamil and Fatima (2018) conducted a study to translate the CCS into Urdu and establish its psychometric properties. The sample was drawn from several educational institutes, and the study demonstrated good alpha reliabilities, strong item-to-item correlations, and good correlations between subscale scores and the full-scale score. Additionally, moderate to low inter-scale correlations further strengthened the psychometric properties of the scale. However, this study also did not examine the factorial validity of the CCS.

In Pakistan, the CCS was tested with an incarcerated population (Ishfaq & Kamal, 2023). However, the psychometric properties of the instrument proved to be questionable, as the CCS showed corrected item-total correlations between 0.15 and 0.49 and lacked internal consistency. Similarly, confirmatory factor analysis showed that the CCS had some items with low factor loadings.

In contrast, in a recent study in Europe conducted with Romanian inmates, the CCS demonstrated good validity and reliability (Dina et al., 2023). The results supported the CCS’s ability to reliably measure criminogenic thinking and its correlation with personality traits and criminal behavior, confirming its utility in assessing cognitive patterns related to criminality. Similarly, this study did not examine the factorial structure of the CCS.

Finally, in Portugal, Santos (2021) conducted the first study with the CCS in a community sample of adults. The CCS demonstrated a lack of theoretical and psychometric support for the five-factor model described by Tangney et al. (2012), suggesting a unidimensional version with 16 items that showed satisfactory psychometric properties (Santos, 2021). Regarding internal consistency, the 16-item scale solution showed α = 0.76, indicating adequate reliability. The positive relationship between criminogenic beliefs and antisocial traits demonstrated good indicators of the CCS’s convergent validity. For divergent validity, the CCS demonstrated, as expected, negative correlations with Social and Emotional Competencies (Santos, 2021). 

Therefore, there is limited research regarding the CCS factor validity, and in Portugal, the CCS’s psychometric properties have only been tested for the general population (Santos, 2021). Due to the lack of validated instruments specifically designed to assess criminogenic cognitions within incarcerated populations, there is a critical need for reliable and culturally appropriate tools to evaluate inmates’ progress in intervention programs. Given the importance of criminogenic cognitions for recidivism, it is essential to conduct a comprehensive examination of the CCS’s psychometric properties for the Portuguese inmate population. To our knowledge, this is the first study to explore the CCS factor structure in a forensic sample in Europe.

The current study represents the first examination of the psychometric properties of the CCS within a population of male inmates in Portugal. While this may be considered a limitation, it is important to note that male inmates represent 93% of the prison population. This study aimed to explore the validity and reliability of the factor structure with a sample of male inmates in Portugal. Specifically, we used exploratory factor analysis and assessed the convergent validity with measures of the antisocial spectrum, moral disengagement, and self-control, analysing the sensitivity of individual items as well as the total scale and its dimensions, and measuring internal consistency with the omega coefficient, composite reliability, and coefficient H.

## 2. Methods

### 2.1. Participants

This study included 364 male inmates (*M*_age_ = 37.88, *SD* = 10.88) convicted of crimes perpetrated after the age of 16 years. They were selected from 16 Portuguese prisons across the central and north of Portugal. On average, participants had 8.63 years of education (*SD* = 3.01) and were mainly single (65%, *n* = 236). Most of the sample were from the ethnic majority group (78.7%, *n* = 281) and were of Portuguese nationality (89.7%, *n* = 321) (see Table 1). Further sociodemographic characteristics are detailed in Table 1.

### 2.2. Instruments

#### 2.2.1. Criminogenic Cognitions Scale (CCS)

The multidimensional CCS self-report measure assesses criminogenic cognitions with 25 items grouped into five dimensions: (a) Notions of Entitlement (e.g., “When I want something, I expect people to provide/do it”); (b) Failure to Accept Responsibility (e.g., “I feel that what happens in my life is determined by powerful people”); (c) Short-Term Orientation (e.g., “The future is unpredictable, and therefore it makes no sense to plan/make plans”); (d) Insensitivity to the Impact of Crime (e.g., “Normally, over time, victims of crimes can overcome them”); and (e) Negative Attitudes Toward Authority (e.g., “The majority of police/guards abuse their power”).

Participants rate their level of agreement with each item, using a 4-point Likert scale (1 = *Strongly disagree* to 4 = *Strongly agree*) (Tangney et al., 2012). The original (Tangney et al., 2012) and Portuguese (Santos, 2021) versions of the CCS—the latter tested in a community sample—showed good psychometric proprieties. In the original version (Tangney et al., 2012), this instrument showed good internal consistency for the total scale (α = 0.81).

#### 2.2.2. Civic Moral Disengagement (CMD)

The CMD instrument (Caprara et al., 2009) assesses acts that violate ethical codes, with 32 items grouped into the eight mechanisms of moral disengagement identified by Bandura et al. (1996): (a) Moral Justification (e.g., “For the advance of science, it is lawful to use humans as ‘guinea pigs’, even in high risk experiments”); (b) Euphemistic Labelling (e.g., “Drawing graffiti on walls is the expression of a ‘creative spirit’”); (c) Advantageous Comparison (e.g., “Given the widespread corruption in society, one cannot disapprove of those who pay for favors”); (d) Displacement of Responsibility (e.g., “When traffic moves quickly, drivers who exceed the speed limit in order to keep up should not be fined”); (e) Diffusion of Responsibility (e.g., “Employees are never responsible for executing the illegal decisions of their bosses”); (f) Distorting Consequences (e.g., “Evading taxes cannot be considered reprehensible considering the squandering of public money”); (g) Attribution of Blame (e.g., “Victims generally have trouble staying out of harm’s way”); and (h) Dehumanization (e.g., “Some people are real disasters”) (Caprara et al., 2009). Despite assessing the eight mechanisms of moral disengagement, the original version of the CMD instrument appeared to have a unidimensional structure.

Participants rate their level of agreement with each item, using a 5-point Likert scale (1 = *Strongly disagree* to 5 = *Strongly agree*) (Caprara et al., 2009). The original (Caprara et al., 2009) and Portuguese (Camelo, 2022) versions of the CMD instrument showed adequate psychometric proprieties. In the current study, this instrument showed appropriate internal consistency (α = 0.91).

#### 2.2.3. Predictive Antisocial Spectrum Questionnaire (PASQ)

The PASQ is a self-report questionnaire that assesses key antisocial traits in forensic populations, with 10 items grouped into two dimensions: (a) Impulsivity/Irresponsibility (e.g., “If I need to take risks, I take them, even if it affects my safety”), which integrates disregard for consequences and acting without thinking, and (b) Interpersonal Relationships (e.g., “I have already gotten into trouble for risking my safety or that of others”), which is the ability to relate to others (Neves, 2023). Participants rate their level of agreement with each item, using a 4-point Likert scale (1 = *Strongly disagree* to 4 = *Strongly agree*), from the beginning of their adolescence (before 15 years old). The instrument was developed in Portugal, and the 10-item version has shown good psychometric proprieties (Neves, 2023). In the current study, this instrument also showed appropriate internal consistency (αs = 0.69–0.77).

#### 2.2.4. Brief Self-Control Scale (BSCS)

The BSCS is a self-report scale that evaluates self-control skills with 13 items (Pechorro et al., 2021). This instrument assesses specific features of self-control, namely self-discipline, deliberate/non-impulsive action, healthy habits, work ethic, and reliability. Participants rate their level of agreement with each item, using a 5-point Likert scale (1 = *Not at all like me* to 5 = *Quite like me*). Higher scores represent better self-control skills. The original version, developed in the United States among male inmates, showed good psychometric properties (Tangney et al., 2004). In Portugal, when administered to forensic samples, the BSCS showed appropriated psychometric properties (Pechorro et al., 2021). In the current study, this instrument also demonstrated appropriate internal consistency (α = 0.69).

#### 2.2.5. Procedure and Analytic Strategy

The current study was part of the research project called *Assessment for Effective Interventions: Reducing the Risk of Criminal Recidivism and Social Marginalization*. Ethical approval was granted by the ISPA Ethics Committee (I/029/01/2020). To conduct data collection, authorization was required by the General Direction of Prison and Probation Services from the Ministry of Justice (GDPPS–MJ), which allowed us to assess up to 10% of the inmates in each prison. The data collection procedure, including dates, methodology, and participant selection, was coordinated with each prison director via email and/or telephone. The exclusion criteria included all factors that could affect an inmate’s ability to consent or understand the aim of the study and the questionnaires (e.g., severe psychopathology, inability to read and comprehend). The sample was recruited from prisons in the metropolitan area of Lisbon (Sintra, Lisboa, Linhó, Carregueira, Montijo, Caxias, Torres Novas) and other prisons in the center (Guarda, Coimbra, Aveiro, Leiria, Leira Jovens) and north (Paços de Ferreira, Braga, Porto, Santa Cruz do Bispo) regions of Portugal.

Participants were randomly selected by justice professionals with a premade list of the prisons, and the questionnaires were administered in small groups of participants (six to ten inmates at a time). The administration of the paper-and-pencil Portuguese questionnaires was supervised by two to four members of the research team in designated areas inside the prisons (e.g., library, school, visiting rooms).

The analyses were performed with IBM’s SPSS Statistics (Version 28) and in the FACTOR program. FACTOR was used to explore the psychometric properties of the scale and to identify its factorial structure, while SPSS was employed for the remaining analyses. The exploratory factor analyses were performed, with the robust minimum rank factor analysis as the extraction procedure. The proamin method was used in the rotation procedure. The Kaiser–Meyer–Olkin (KMO) measure and Barlett’s test were used to evaluate correlations between variables. The number of factors was determined by loading values above 0.30 and 0.40. All the items that saturated simultaneously in two factors were removed. Reliability was assessed through internal consistency, using the omega coefficient, composite reliability, and the coefficient H.

Convergent validity between the CCS, the PASQ, CMD, and BSCS subscales were analysed with Pearson correlations. The sensitivity of the items, total scale, and its dimensions were examined through univariate statistics (e.g., means, minimum and maximum, medians, percentiles, and standard deviations).

## 3. Results

### 3.1. Exploratory Factor Analysis

In order to explore the underlying structure of the CCS, an exploratory factor analysis was conducted on the dataset. The primary objective was to identify the latent factors that best represent the CCS structure. The KMO measure and Bartlett’s test results showed good indicators of correlation between variables (KMO = 0.82; Bartlett’s test, χ^2^ = 1841.2, *df* = 105, *p* < 0.001). First, a five-factor structure was tested, since the structure originally proposed by Tangney et al. (2012) was comprised of five factors. However, the five-factor structure proved to be unsustainable, and instead, a two- or three-factor solution was recommended (Timmerman & Lorenzo-Seva, 2011). A 15-item, two-factor structure emerged, with the communalities above 0.30 and the factor loadings above 0.40. Table 2 displays the factor loadings and communalities.

Regarding the two identified factors, Factor 1 comprised eleven items (1, 3, 4, 5, 7, 8, 18, 22, 23, 24, 25), while Factor 2 comprised four items (9, 13, 14, 21). The designations of each factor were based on the content of their respective items. Factor 1 was named “Short-Term Orientation and Responsibility Evasion”, since it included the five items of the original “Short-Term Orientation” dimension proposed by Tangney et al. (2012) and included items related to the denial of responsibility for one’s actions. On the other hand, Factor 2 was labelled “Authority Resistance”, since its items reflect negative attitudes toward authority. The communalities were larger than 0.35, and the factor loadings ranged between 0.44 and 0.94. The two-factor finding explained 64.42% of the variance, with the first dimension named Short-Term Orientation and Responsibility Evasion explaining 47.69% of the variance and Authority Resistance explaining 16.73% of the variance.

### 3.2. Convergent Validity

In the assessment of convergent validity (Table 3), correlations between the CCS and the PASQ, CMD, and BSCS were calculated and found to be statistically significant. The strongest correlation was between the CCS total score and the CMD instrument (*r* = 0.69). Positive and moderate correlations were observed between the Irresponsibility/Impulsivity dimension of the PASQ and both the CCS total score (*r* = 0.44) and the Short-Term Orientation and Responsibility Evasion factor of the CCS (*r* = 0.41). The correlations between the CCS and the CMD instrument were also statistically significant, ranging from moderate to large effect sizes (Cohen, 1988). For the BSCS, although the correlations were significant, they were negative and displayed a small effect size.

### 3.3. Reliability: Internal Consistency

The reliability of the CCS was assessed through internal consistency, using the omega coefficient, composite reliability, and the coefficient H. Cronbach’s alpha was not employed because one of the dimensions—the Authority Resistance dimension—contained only four items, making Cronbach’s alpha less suitable for scales with a small number of items (Kalkbrenner, 2023). Instead, Omega coefficient was preferred, as it does not assume tau-equivalence and accounts for heterogeneous factor loadings, allowing for a more accurate estimation of internal consistency reliability in scales with limited items (Dunn et al., 2014). The values obtained can be considered acceptable (see Table 4), ranging 0.60–0.95 (Flora, 2020).

### 3.4. Sensitivity Analysis

The minimum and maximum values recorded in the responses to each item demonstrated that, for all items, each of these alternatives (1 = *Totally disagree*; 4 = *Totally agree*) was chosen by at least one participant (Table 5). The total scale showed a mean value of 2.06 (*SD* = 1.49), and scores ranged between 1.40 in the 10th percentile and 2.67 in the 90th percentile. For each factor, the mean values ranged between 1.79 (Short-Term Orientation and Responsibility Evasion) and 2.79 (Authority Resistance).

Table 6 displays the scores for the total scale and dimensions as percentiles (10, 20, 30, 40, 50, 60, 70, 80, 90), showing that the data were distributed along the total scale and the dimensions, thereby suggesting that the scale is sensitive and capable of distinguish different performance levels.

## 4. Discussion

To our knowledge, this is the first study to test the factor structure of the CCS with European inmates. Assessing psychological measures in prison populations can be challenging, as screening tools may overestimate disorder prevalence due to non-specific items and lack of validation in this context (Fazel et al., 2016). This concern is heightened by the fact that many diagnostic instruments commonly used in correctional settings were originally developed for general populations and have not been properly validated for incarcerated individuals, leading to potential inaccuracies. Although Tangney et al. (2012) provided considerable evidence of the scale’s reliability and validity, the authors did not examine its factor structure. Specifically, no exploratory factor analysis was performed to empirically verify the grouping of items into latent factors. Therefore, the aim of this study was to assess the psychometric properties of the CCS with a sample of Portuguese inmates. Aspects such as factor structure, item and dimension sensitivity, internal consistency, and convergent validity were thoroughly examined.

We identified a two-factor structure, and after an item-content analysis, the two factors were subsequently named “Short-Term Orientation and Responsibility Evasion” and “Authority Resistance”. A KMO of 0.82 and significant Bartlett’s test values were found, suggesting an appropriate fit (Marôco, 2018). All items demonstrated communalities of *h*^2^ = 0.35 or higher, fulfilling the parameters set by Child (2006), and each item in the scale showed loadings ranging between *λ* = 0.44 and *λ* = 0.94, meeting the criterion established by Field (2018).

In particular, the small sample size was insufficient to be divided into separate sub-samples to perform a confirmatory factor analysis based on the factor structure identified in the exploratory factor analysis conducted in this study. This factor solution substantially differs from that initially proposed by Tangney et al. (2012) for a population with U.S. inmates, which may be explained by several reasons. Firstly, the original study did not empirically assess factorial validity but relied solely on a theoretical conceptualization to propose the five-factor structure. The lack of factorial validity assessment in the original study (Tangney et al., 2012) does not allow for the establishment of more robust, evidence-based knowledge about the factor structure of the CCS, even within the U.S. incarcerated population.

In addition, Ishfaq and Kamal (2023) conducted a Confirmatory Factor Analysis (CFA) to evaluate the CCS five-factor structure initially proposed by Tangney et al. (2012). Their findings suggested that the proposed factor structure exhibited poor construct validity, with suboptimal model fit indices (CFI = 0.65, TLI = 0.58, RMSEA = 0.07). This study highlights the need to examine the CCS factor structure, particularly in non-U.S. samples of inmates.

Moreover, the differences identified in the study conducted in Pakistan suggest that the CCS theoretical dimensions might be influenced by substantial cultural differences. In this regard, the Portuguese justice system is characterized by substantially different legal and cultural aspects compared to the U.S. justice system, which may influence the CCS factor structure and other psychometric properties, making a preliminary exploratory factor analysis (EFA) highly recommended. For instance, Portugal decriminalized drug consumption in 2000 (Rego et al., 2021), whereas the U.S. has a large population incarcerated for crimes related to illegal drug use (Carson & Kluckow, 2024), leading to substantially different prison population profiles. In addition, in Portugal, sentences are considerably shorter compared to the U.S., where only more serious or highly recidivist crimes result in prison sentences (Aebi & Cocco, 2024; Council on Criminal Justice, 2022). Both factors have a relevant impact on labelling effects (e.g., Bernburg et al., 2006) and deviant peer contagion (e.g., Dishion & Tipsord, 2011) phenomena and, probably, on associated criminogenic cognitions.

The first factor was drawn from items that Tangney et al. (2012) identified as part of a Short-Term Orientation dimension, with few additional items from the Failure to Accept Responsibility, Insensitivity to the Impact of Crime, and Notions of Entitlement dimensions. In particular, the items related to entitlement thinking that loaded onto the first factor were also associated with a deviation from personal responsibility and an inability to recognize the consequences of one’s actions.

The dimension “Short-Term Orientation and Responsibility Evasion” aligns with several established theoretical models. For instance, according to Simon’s (1957) theory of bounded rationality, individuals make decisions based on limited information and often prioritize immediate rewards over long-term consequences. This is particularly relevant for criminal behavior, where justice-involved individuals may exhibit a strong preference for short-term gains, neglecting potential future repercussions (Van Gelder et al., 2015). Similarly, Sykes and Matza’s (1957) neutralization theory suggests that individuals engaged in criminal behavior use various cognitive strategies to justify their actions, often by denying responsibility and minimizing potential consequences (cf. Zuber et al., 2016). The relationship between criminogenic cognitions and antisocial behavior can also be linked to Hare’s Psychopathy Theory (Hare, 1991; Magyar et al., 2010), which highlights impulsivity as a core trait of psychopathy. Impulsivity, combined with shallow affect and poor behavioral controls, often leads to a focus on immediate gratification and a disregard for consequences—behaviors that are common among justice-involved populations. These behaviors may be followed by responsibility evasion through neutralization techniques (Kaptein & van Helvoort, 2019; Sykes & Matza, 1957). In other words, these cognitive beliefs may lead to the denial of personal responsibility for acts resulting from a short-term orientation, thereby promoting the maintenance and reinforcement of these criminogenic patterns. For instance, interventions targeting Short-Term Orientation and Responsibility Evasion could focus on enhancing inmates’ decision-making skills, promoting delayed gratification, fostering accountability for one’s actions, and addressing impulsivity.

On the other hand, the “Authority Resistance” factor comprised items from the original dimensions “Failure to Accept Responsibility”, “Insensitivity to the Impact of Crime”, and “Negative Attitudes to Authority”, which may also be representative of an underlying association between these dimensions. In the original study by Tangney et al. (2012), the researchers found that all dimensions of criminogenic cognitions were negatively related with empathic concern and perspective taking, which may explain the possible association with “Insensitivity to the Impact of Crime”. Most of the items of the “Authority Resistance” factor were from the original dimension called “Negative Attitudes to Authority”.

The prominent role of this factor can be understood through various theories that address the relationship between individuals and social control mechanisms. For instance, Hirschi’s (1969) social control theory proposes that weaker bonds to social institutions (e.g., family, school, work) result in less conformity to societal norms and a greater propensity to resist authority. Individuals with weaker social bonds are more likely to challenge and resist authority figures (Jensen, 2011). Similarly, Merton’s (2014) theory of anomie highlights how social structures can lead to a lack of societal norms and values and deviant behavior when individuals experience a disconnect between societal goals and the means available to achieve them. This strain can foster attitudes of resistance toward authorities perceived as perpetuating inequality (Hövermann & Messner, 2019). Inmates may view their actions as a rebellion against a system they consider unjust or illegitimate (Rios, 2017).

The “Authority Resistance” factor may also reflect learned patterns from peers or environments where deviance is normalized, highlighting the need for social skills training and exposure to pro-social role models during incarceration. Therefore, the Authority Resistance factor might result from previous deviant peer contact, labelling effects, and negative encounters with justice authorities (e.g., Bernburg et al., 2006; Dishion & Tipsord, 2011). Addressing Authority Resistance through programs aimed at strengthening pro-social attitudes and improving perceptions of authority figures may reduce defiance and increase compliance with societal norms. Measures of social insertion (e.g., improving education and job opportunities, programs addressing criminogenic needs), together with the promotion of healthy, empathetic, and trustworthy relationships between prison staff and inmates, may challenge criminogenic cognitions related to authority resistance and prevent criminal behaviors. Such interventions may include building trust, promoting positive interactions with authority figures, and developing skills for managing oppositional behavior.

As previously mentioned, both factors comprised items related to “Failure to Accept Responsibility”, which may be indicative of a relationship with the remaining dimensions. Moral disengagement might be an important concept to explain this hypothesis. For instance, the criminogenic dimensions may sustain and reinforce moral disengagement, and consequently, an individual’s involvement in acts that violate moral principles and social norms without feeling guilt and shame (Bandura, 2016; Walters, 2020).

Regarding reliability, the analysis of the internal consistency measured by the omega coefficient, composite reliability, and coefficient H revealed acceptable values above 0.60 for the total scale and its factors; however, Factor 2 exhibited an omega coefficient below 0.70 (Finch et al., 2016). These findings are consistent with Tangney et al. (2012), who also reported satisfactory internal consistency for the CCS, though the authors did not examine these specific reliability indicators. In addition, the convergent validity of the CCS with measures of the antisocial spectrum and moral disengagement mostly revealed low to moderate correlations, similar to the patterns identified in the original study. Larger correlations were found between the CCS and moral disengagement, which was expected, given that both criminogenic cognitions and moral disengagement share a set of psychological mechanisms that reduce personal accountability and enable offenders to perceive antisocial behaviors as acceptable (Shulman et al., 2011). These mechanisms include diffusion of responsibility, downplaying the harm caused to the victim, and minimizing the consequences of antisocial acts. Moreover, we found a statistically significant negative correlation between CCS and self-control, which is also consistent with previous research (DeLisi et al., 2003; Tangney et al., 2012; Walters, 2017c).

## 5. Conclusions

This study represents one of the first attempts to investigate the psychometric properties of the CCS among inmates. It was the first to examine the factorial structure of the CCS in a European inmate population, as well as the first to evaluate the psychometric properties of the CCS with an incarcerated Portuguese sample. In a nutshell, the findings from the exploratory factor analysis, convergent validity, reliability, and sensitivity analysis suggest that the CCS is a promising tool for the assessment of criminogenic cognitions in Portuguese inmates.

Future studies should address some key limitations identified in this research. First, our sample was limited to incarcerated males, restricting the generalizability of the findings. To overcome this, future research should include female inmates and individuals serving community-based sentences to ensure broader applicability across different populations. Additionally, the relatively small sample size may have limited the statistical power of the results, and larger, more diverse samples are recommended for more robust conclusions. In particular, the small sample size was insufficient to be divided into separate subsamples to perform a confirmatory factor analysis based on the factor structure identified in the exploratory factor analysis conducted in this study. Our reliance on self-reported measures introduced the potential for social desirability bias, which could be mitigated by incorporating objective assessments or cross-referencing with other data sources. Finally, this study did not evaluate important psychometric properties such as criterion validity or test–retest reliability. Future research should incorporate these assessments, alongside confirmatory factor analysis, to ensure the rigor and reliability of the measures used.

Overall, our findings suggest that the CCS is a practical and effective tool for assessing criminogenic cognitions. By identifying and addressing criminogenic cognitions, the CCS can contribute to more targeted and effective interventions aimed at reducing criminal behavior and supporting rehabilitation efforts. Furthermore, the CCS can be crucial for assessing inmates’ progression in intervention programs, offering a valuable framework for addressing specific criminogenic needs that lead to recidivism. This allows for the assessment of inmates’ progress and the implementation of appropriate program measures, ultimately enhancing rehabilitation outcomes.

In conclusion, this study has provided a fundamental assessment of the CCS within a specific context and has highlighted the importance of ongoing research to refine and validate the instrument. Expanding the scope of research to encompass diverse populations and contexts will be crucial for enhancing the CCS’s applicability and effectiveness as a tool for understanding and addressing criminogenic cognitions across different inmate groups and other justice settings.

## Figures and Tables

**Table 1:** Sociodemographic characteristics of the sample.

Variables		*M*	*SD*
Age		37.88	10.88
Education		8.63	3.01
Minority Group	No	281	78.7%
	Yes	76	21.3%
Nationality	Portuguese	321	89.7%
	Spanish	1	0.3%
	Brazilian	9	2.5%
	Angolan	6	1.7%
	Another	21	5.9%
Marital status	Single	236	65%
	Unmarried living together	48	13.2%
	Married	30	8.3%
	Divorced/separated	48	13.2%
	Widower	1	0.3%
Parenthood	No	130	35.7%
	Yes	234	64.3%
Recidivism	No	169	48.2%
	Yes	182	51.9%

Abbreviations: *M*, mean; *SD*, standard deviation; *N*, total of participants; %, percentage of participants.

**Table 2:** Results of the factor analysis, loadings, and communalities of the CCS.

Items	Short-Term Orientation and Responsibility Evasion (F1)	Authority Resistance (F2)	Communalities
7. A theft is all right as long as the victim is not physically injured.	**0.78**	0.06	0.84
18. Why plan to save for something if you can have it now?	**0.76**	−0.10	0.78
8. Even though I got caught, it was still worth the risk.	**0.72**	−0.13	0.69
22. I am just a “born criminal”.	**0.68**	−0.12	0.73
23. I deserve more than other people.	**0.64**	−0.15	0.60
24. I think it is better to enjoy today than worry about tomorrow.	**0.60**	0.06	0.66
4. My crime(s) did not really harm anyone.	**0.58**	0.11	0.63
3. The future is unpredictable and there is no point planning for it.	**0.57**	0.11	0.67
5. I feel like what happens in my life is mostly determined by powerful people.	**0.51**	0.12	0.71
1. When I want something, I expect people to deliver.	**0.47**	0.13	0.37
25. I do not like to be tied down to a regular work schedule.	**0.44**	0.19	0.67
13. Most police officers/guards abuse their power.	−0.03	**0.94**	1.00
21. People in positions of authority generally take advantage of others.	0.06	**0.67**	0.61
9. Because of my history I get blamed for a lot of things I did not do.	0.23	**0.46**	0.54
14. Society makes too big of a deal about my crime(s).	−0.20	**0.45**	0.35

**Table 3:** Convergent validity.

	PASQ Total	PASQ Irresponsibility/Impulsivity	PASQ Interpersonal Relationship	CMD	BSCS
CCS Total	0.39 **	0.44 **	0.26 **	0.69 **	−0.43 **
Short-Term Orientation and Responsibility Evasion	0.33 **	0.41 **	0.20 **	0.68 **	−0.42 **
Authority Resistance	0.33 **	0.28 **	0.27 **	0.33 **	−0.20 **

Note. ** *p* < 0.01.

**Table 4:** Internal Consistency.

Factor	Number of Items	Omega Coefficient	Composite Reliability	Coefficient H
Short-Term Orientation and Responsibility Evasion	11	0.77	0.87	0.89
Authority Resistance	4	0.60	0.74	0.90
CCS Total	15	0.76	0.91	0.95

**Table 5:** Distributional characteristics of the items, dimensions, and the total scale.

Item Number	Mdn	Mean	SD	Minimum	Maximum
Item 1	2	1.83	1.98	1	4
Item 3	2	1.96	2.11	1	4
Item 4	1	1.82	2.11	1	4
Item 5	1	1.85	2.08	1	4
Item 7	1	1.48	1.89	1	4
Item 8	1	1.38	1.81	1	4
Item 18	1	1.77	1.92	1	4
Item 22	1	1.36	1.77	1	4
Item 23	1	1.53	1.79	1	4
Item 24	2	2.09	2.09	1	4
Item 25	3	2.58	2.19	1	4
CCS Short-Term Orientation and Responsibility Evasion	1.73	1.79	1.55	1	3.73
Item 9	3	2.53	2.18	1	4
Item 13	3	2.96	2.01	1	4
Item 14	3	2.85	2.02	1	4
Item 21	3	2.84	1.98	1	4
CCS Authority Resistance	2.75	2.79	1.70	1	4
CCS Total	2	2.06	1.49	1	3.80

**Table 6:** Percentile scores of Criminogenic Cognition Scale dimensions.

Percentiles	CCS Total	CCS Short-Term Orientation and Responsibility Evasion	CCS Authority Resistance
10	1.40	1.09	1.75
20	1.67	1.27	2.25
30	1.80	1.45	2.50
40	1.87	1.55	2.50
50	2.00	1.73	2.75
60	2.13	1.91	3.00
70	2.33	2.00	3.25
80	2.47	2.18	3.50
90	2.67	2.55	3.75

## Data Availability

The datasets presented in this article are not readily available because the data are part of an ongoing study. Requests to access the datasets should be directed to the corresponding author.
